# Supplementary material for: Operationalization and Reporting Practices in Manuscripts Addressing Gender Differences in Biomedical Research: A Cross-Sectional Bibliographical Study
Source: Int J Environ Res Public Health. 2022 Nov 1;19(21):14299. doi: 10.3390/ijerph192114299 (PMC9653596; doi:10.3390/ijerph192114299)
Supplement: Supplementary file 1 [file ijerph-19-14299-s001.zip › ijerph-1935602-supplementary.pdf]

General

Case number

Title

First and last author full names

Inferred gender first author

Inferred gender last author

Discipline

Discipline Group

Journal

Abstract

A1 Is the abstract structured?

A2 Is the objective of the manuscript gender specific research?

A3 Are gender differences mentioned in the introduction/background?

A4 Do the methods/results detail gender identity of the included subjects?

A5 Are results reported in gender disaggregated manner?

A6 Are the conclusions addressing gender differences?

A7 If A6=1, are the consequences of this difference addressed?

Introduction

I1 Is gender explicitly defined?

I2 Is background information about gender differences reported?

I3 Is preliminary data about the studied gender difference reported?

I4 Do the authors state a hypothesis about the expected difference?

I5 Is the need for gender specific analysis substantiated?

Methods

M1 Is any attribution of gender described?

- How is gender attributed?

M2 Was any dimension of gender addressed?

- Which dimension of gender was addressed?

M3 Was gender taken into consideration in the recruiting process?

M4 Are details given towards the statistical ability to capture gender differences with the included sample? (e.g. power calculation)

M5 Are gender factors relevant to the access to the study mentioned?

M6 Are gender factors relevant to the functioning of the study/product to be tested/measure mentioned?

M7 Is anything mentioned about gender distribution of the research team?

M8 Was the analysis disaggregated by gender?

M9 Have any gender specific ethical aspects been taken into account?

Results

R1 Are the included numbers reported by gender (identity)?

R2 Is the analyzed data disaggregated by gender (identity)?

R3 Are results reported in gender-disaggregated manner even if no difference was identified?

R4 Are drop-outs, withdrawals, outliers, loss-to-follow up reported by gender (identity)?

R5 Are possible gender specific confounders (explicitly) corrected for?

R6 Are intersectional analyses included?

- If R6 yes, which aspects of intersectionality are included?

R7 Are gender differences represented in tables, figures or graphs?

Discussion

D1 Are any gender specific considerations regarding the extent to which data can be generalized included? And considerations to which the gender difference can be generalized?

D2 Are the reasons for potential gender differences discussed?

D3 Are the implications of gender differences discussed?

D4 Are actionable consequences of gender differences discussed?

D5 Are gender specific limitations due to lack of generalizability, differences in participation, etc. discussed?
